# Cannabidiol (CBD) in the Self-Treatment of Depression-Exploratory Study and a New Phenomenon of Concern for Psychiatrists

**DOI:** 10.3389/fpsyt.2022.837946

**Published:** 2022-03-22

**Authors:** Gniewko Wieckiewicz, Iga Stokłosa, Maciej Stokłosa, Piotr Gorczyca, Robert Pudlo

**Affiliations:** Department and Clinic of Psychiatry, Medical University of Silesia, Tarnowskie Góry, Poland

**Keywords:** cannabidiol, cannabis, marihuana, depression, self-treatment

## Abstract

Cannabis sativa, whose flowers are also known as marijuana or marihuana, is a recreational plant that contains many chemicals that are constantly being studied by scientists around the world. One of these substances is cannabidiol (CBD), which has gained widespread popularity on the internet as a cure for mental health problems, leading many people to use CBD to self-treat depression and anxiety. This article presents an exploratory cohort study (*n* = 90) of a group of people aged 16–69 using CBD to self-heal depression symptoms. The survey included basic sociodemographic questionnaire and validated Hospital Depression and Anxiety Scale. And was distributed *via* the Internet. The results were statistically analyzed. High school degree was the most commonly held education (46%), large city was the most popular place of living (33%) and majority of the respondents have a full-time job (53%). Only 19% of the respondents consult their doctor or pharmacists about taking CBD. On the group of psychiatric patients, only 49% of respondents tell their psychiatrist about using the compound. Psychiatrists should be aware of CBD use in their patients during their daily practice, as CBD use can be found within people from all walks of life, and due to public interest, there is a need for education and research on the efficacy and safety of CBD use for mental disorders.

## Introduction

Cannabis sativa, commonly known as marijuana or marihuana, is a plant with psychoactive properties used primarily for recreational purposes. However, in recent years, numerous studies have been conducted that have found its beneficial effects in the treatment of many diseases (1). Marijuana-derived compounds, known for their antioxidant, anti-inflammatory, and antinecrotic properties, are considered promising agents that are increasingly used in research related to Parkinson's disease, epilepsy, depression, anxiety disorders, and schizophrenia, as well as in the treatment of chronic pain (2–4). The substances contained in marijuana are called cannabinoid com-pounds. The most potent constituent of cannabis is natural tetrahydrocannabinol (THC), which is responsible for the psychoactive properties of marijuana (1). Among other compounds, one is especially notable–cannabidiol (CBD), a non-psychoactive compound which could be useful in depression treatment, as the studies have demon-strated the activity of CBD as a partial agonist of 5HT1a serotonin receptors, which could be beneficial in the treatment of depression and anxiety by using this substance, but this still requires extensive research (5).

CBD appears to be relatively safe substance in preliminary studies, but there are several side effects that should be mentioned. CBD is one of the better tolerated substances compared to THC, mainly due to its lower addictive potential (6). In the available literature, the adverse effects described mainly refer to studies in animal models and depend on the dose taken and the duration of use. The use of CBD in animals resulted in the development of drug toxicity, increased fetal mortality, liver cell damage, inhibition of spermatogenesis, and hypotension, but it should be mentioned that the doses used in animals were above the recommended amounts for humans (7). The most common side effects reported in studies of cannabinoid use for epilepsy or psychotic disorders were fatigue, diarrhea, and appetite disturbances (8). Other side effects reported after CBD use included vomiting, insomnia, and hepatologic disorders. Nonetheless, in certain conditions CBD could be dangerous, as it is metabolized in the liver with the involvement of CYP3A4, which affects its interactions with many drugs that are also processed with the involvement of this enzyme system (including anti-fungals, clarithromycin, or rifampicin) (7).

The public is very interested in natural methods to treat depression. Scientists are focusing on the study of dimethyltryptamine (DMT), a psychedelic substance found in many plants, and psilocybin, a psychedelic that occurs naturally in mushrooms such as psilocybin cubensis (9). The popularity of CBD in the treatment of depression is as great in society as the popularity of the use of DMT or psilocybin-on October 15, 2021, the Google search engine returns 6,370,000 results for the term “CBD depression treatment,” and information on this topic can be found on such well-known websites as the New York Times or Forbes (10, 11). Despite the great popularity that the use of CBD for depression enjoys on the Internet, in our opinion, the scientific data on the efficacy and safety of this substance in the treatment of depression remain sparse. It is not difficult to find groups on social media (e.g., Facebook) where experiences are shared about the use of CBD for self-care for mental health and where people (often without medical training) recommend certain products from the Internet along with dosage. Self-care for mental health has its limits, and that is when patients turn to supplements and products purchased online without the knowledge of their doctor, as this is potentially dangerous. There are documented over-the-counter uses of St. John's wort in combination with serotonin reuptake inhibitors that resulted in the development of serotonin syndrome (12). Because we do not know much about CBD, we believe that people who use CBD to self-medicate should be closely monitored. We were unable to find appropriate studies describing this phenomenon in any disease, although previous literature suggests that self-medication with CBD exists for chronic pain, anxiety, and depression (13). In our opinion, the availability of CBD on the retail market is disproportionate to the number of scientific reports on the efficacy and safety of CBD, because in many European countries such as Austria, Spain, Sweden, Germany or France you can easily buy CBD legally (14). This situation is potentially dangerous from a medical perspective for both patients and medical staff, as people risk potentially treacherous intoxication by searching social media for unverified data on the ingestion of rather unknown substances. Therefore, as a group of psychiatrists, we decided to investigate the problematic phenomenon of using CBD to self-treat depressive symptoms, as it is important to learn more about the people who choose to do so. We aimed to explore the basic demographic and epidemiological characteristics of people who use CBD to self-treat their depressive disorders and to demonstrate the fact that this phenomenon exists. The study was exploratory in nature, therefore we did not rise any particular research questions.

## Materials and Methods

The study was designed by psychiatrists from the Department of Psychiatry at the Medical University of Silesia in Katowice and was conducted according to the guide-lines of the Declaration of Helsinki and Good Clinical Practice. It included 23 questions in Polish in the areas of: general sociodemographic parameters, general psychiatric interview of patients, questions related to CBD intake: frequency, dosage and form of consumption, improvement of wellbeing after CBD intake and additionally included the Hospital Anxiety and Depression Scale (HADS) questionnaire. The HADS is one of the most widely used self-assessment questionnaires for screening anxiety/depression symptoms and focuses mainly on the cognitive and psychological aspects. It is used in both the general medical population and the healthy population. The HADS consists of a total of 14 items on 2 separate subscales: Anxiety (HADS-A) and Depression (HADS-D), and the total score ranges from 0 to 42 points. Currently, the categorization system includes several groups: 0–7, normal; 8–10, mild; 11–15, moderate; over 16, severe (15). The survey was uploaded to the Internet via Google Forms, Google's original online survey tool. The form consisted of 5 separate pages-consent to the study, questions about demographic data, questions about previous psychiatric treatment, questions about CBD use, and the HADS questionnaire. Data were collected via Facebook from August 27, 2021 to September 16, 2021. We asked administrators of depression, mental illness, and CBD use groups and websites to help us collect data, and they actively provided a link to the form on their websites, therefore we could not estimate the amount of people who received the link to the survey. Incomplete questionnaires were rejected. To ensure complete anonymity, as marihuana is still generally a taboo subject, no personal or contact information was collected, including email addresses or IP addresses that would identify respondents. For that reason, we had to avoid sampling methods that would be normally used in such study. We had to avoid using data collection enhancement methods, as they would require us to use more complex technical methods that would not allow data anonymization. Participation in the survey was voluntary, respondents were informed of the purpose of the survey and were required to answer in the affirmative to the first question “I use CBD oil to improve symptoms of depression and agree to participate in this anonymous study (or as a minor, I have the consent of my legal guardian to participate),” otherwise they were not given access to the questionnaire. Ninety seven responses were collected, of which 7 subjects, after reading the manual, did not agree to submit their anonymous responses to analysis.

The collected data were analyzed using STATISTICA 13.0 software (StatSoft, Kraków, Poland). Qualitative variables were tested using the chi-square test. The Shapiro-Wilk test was used to check whether quantitative variables conformed to the normal distribution. The test revealed that not all variables conformed to the normal distribution. In case of non-normal distribution, Mann-Whitney U test was used to compare two independent groups, while Kruskal-Wallis test was used to compare multiple independent samples. Spearman's rank order correlation test was used to test the relationship between the variables. Statistical significance was assumed at *p* < 0.05.

## Results

We collected 90 correctly completed questionnaires from the respondents. The study comprised a group of males and females of comparable size who did not differ significantly in age, education and type of occupation. One person reported being non-binary and was excluded from the statistical analysis. The youngest respondent was 16 years old and the oldest was 69 years old. High school degree was the most commonly held education (46%), large city was the most popular place of living (33%) and majority of the respondents have a full-time job (53%). Majority of the respondents claim that they either trust or probably trust the psychiatrists. The detailed characteristics of the study population are shown in Table 1. There is no difference in trust in psychiatrists between the groups. Respondents' place of residence differs between gender groups, but the significance level is borderline, which could be tested if a larger sample of respondents were used.

**Table 1:** Sample characteristics.

		**All**	**Male**	**Female**
***n***	**90**	**42**	**48**
**%**	**100%**	**46,7%**	**53,3%**
	**Average age**	33,7	32,9	34,5
Education	High school degree	46%	43%	50%
	Vocational school	9%	7%	8%
	Higher bachelor's degree or equivalent	14%	14%	15%
	Higher masters or equivalent	30%	33%	27%
	Doctorate or higher academic title	1%	2%	0%
Place of living	Village	21%	26%	17%
	Small town (up to 50,000 inhabitants)	21%	12%	29%
	Medium-sized city (from 50,000 to 200,000 inhabitants)	25%	19%	29%
	A large city (over 200,000 inhabitants)	33%	43%	25%
Type of work	Full-time job	53%	50%	54%
	Entrepreneur	15%	21%	10%
	Another form of employment	10%	10%	10%
	Unemployed	7%	10%	4%
	Seasonal job	7%	2%	10%
	Student	5%	2%	8%
	Pupil	3%	5%	2%
Do you trust psychiatrists?	Definitely not	6%	2%	8%
	Probably not	29%	36%	23%
	Probably yes	44%	43%	46%
	Yes	21%	19%	23%

Majority of the respondents were or still are treated by a psychiatrist (55%) and started using CBD for depressed mood (69%). The most commonly consumed other psychoactive substance was caffeine (47%). Only 19% of respondents consulted a doctor or pharmacists about taking CBD, and most respondents (59%) consume CBD daily. Majority of the respondents (57%) are currently under the supervision of a psychiatrist and a little over half (51%) do not tell their psychiatrists about their use of CBD. Majority of respondents said they felt better after CBD treatment (86%). The detailed characteristics of CBD use for self-treatment of mental disorders are shown in Table 2.

**Table 2:** CBD consumption characteristics.

			**All**	**Male**	**Female**
***n***			**90**	**42**	**48**
**%**			**100%**	**46,7%**	**53,3%**
Have you ever been diagnosed / diagnosed or treated / treated by a psychiatrist?		Yes	55%	40%	67%
		No	45%	60%	33%
Answers only from the group of people who have ever been diagnosed or treated by a psychiatrist (*n* = 50)	Why did you receive psychiatric treatment?	Anxiety disorders	76%	94%	66%
		Depression	72%	76%	69%
		Insomnia	36%	35%	37%
		Personality disorder	16%	18%	13%
		Addiction	14%	18%	13%
		Bipolar affective disorder	4%	0%	6%
		Schizophrenia	4%	6%	3%
	Does your psychiatrist know you are taking CBD?	Yes	49%	47%	50%
		No	51%	53%	50%
	Are you undergoing psychiatric treatment right now?	Yes	57%	41%	66%
		No	43%	59%	34%
Why did you start to use CBD?		Depressed mood	69%	74%	65%
		Anxiety	62%	60%	65%
		Insomnia	58%	45%	69%
		No motivation	48%	38%	56%
		Problems with concentration	40%	33%	46%
		Energy drop	37%	26%	46%
What other psychoactive substances are you using?		Caffeine	47%	55%	40%
		THC	38%	50%	27%
		Nicotine	33%	26%	40%
		Alcohol	21%	33%	10%
		Hallucinogenic substances	7%	7%	6%
		Psychostimulants	2%	0%	4%
		None	29%	19%	38%
Where did you first heard of CBD?		Internet	62%	76%	50%
		Friends	29%	17%	40%
		Family	8%	5%	10%
		Television	1%	2%	0%
Have you consulted CBD consumption with a doctor or a pharmacist?		Yes	19%	14%	23%
		No	81%	86%	77%
Where do you buy CBD most often?		Online shop	66%	67%	65%
		Local store	22%	24%	21%
		Pharmacy	2%	0%	4%
		Friends	10%	10%	10%
What form do you most often consume CBD?		CBD oil	73%	62%	83%
		Hemp drought	26%	38%	15%
		Pure CBD in the form of a spray	1%	0%	2%
How often do you consume CBD?		Every day	59%	60%	58%
		A few times a week	22%	21%	23%
		Several times a month	13%	10%	17%
		Several times a year	4%	7%	2%
		Less often	1%	2%	0%
Do you usually measure the same doses of CBD?		No, I'm not measuring my doses	38%	43%	33%
		Yes, 1–50 milligrams a day	39%	33%	44%
		Yes, 51 to 100 milligrams a day	9%	10%	8%
		Yes, 101 to 150 milligrams a day	9%	10%	8%
		Yes, 151 to 200 milligrams a day	3%	5%	2%
		More than 200 milligrams a day	2%	0%	4%

Women were more likely to be diagnosed or treated by a psychiatrist compared to men (χ^2^ =; 6.19 *p* = 0.01; chi-square test). Among the psychiatric disorders treated, men were significantly more likely to be diagnosed with anxiety disorders (χ^2^ = 4.87; *p* = 0.027; chi-square test). No significant difference was found between genders for the other disorders. Women were significantly more likely than men to take CBD due to insomnia (χ^2^ = 5.07; *p* = 0.024) and energy depletion (χ^2^ = 3.72; *p* = 0.05 (borderline); chi-square test). Men were significantly more likely than women to use THC (χ^2^ = 5; *p* = 0.025) and alcohol (χ^2^ = 7.06; *p* = 0.008 chi-square test).

Men were significantly more likely than women to learn about CBD from the Internet, while women learned from friends and family (χ^2^ = 8.61; *p* = 0.04 chi-square test). Respondents most frequently purchased CBD from online stores, while the most common form of CBD consumption was CBD oil, which was significantly more frequently consumed by women (χ^2^ = 7.12; *p* = 0.03 chi-square test).

The older the individuals were, the more frequently CBD was consumed (*r* = 0.32; *p* < 0.002; Spearman's rank-order correlation). There were no significant differences between genders in frequency of use, amount of dose taken, or reported improvement in wellbeing after taking CBD.

Majority of the respondents (53%) claim that CBD made them feel overall better and 88% of the respondents would more likely take CBD than a prescription drug from a psychiatrist. Table 3 shows detailed psychiatric outcome analysis for respondents using CBD.

**Table 3:** Assessment of CBD effects on mental health of respondents.

		**All**	**Male**	**Female**
***n***		**90**	**42,00**	**48,00**
**%**		**100%**	**46,7%**	**53,3%**
Did CBD make you feel overall better?	Definitely not	6%	7%	4%
	Probably not	8%	10%	6%
	Probably yes	33%	31%	35%
	Definitely yes	53%	52%	54%
With your current knowledge, would you be more likely to take CBD or prescription drugs from a psychiatrist?	Prescription drugs	12%	64%	36%
	CBD	88%	44%	56%
HADS–average result	Anxiety	10,43	9,17	11,58
	Depression	8,04	8	8,16
HADS categories in anxiety subscale	Normal	31%	43%	21%
	Mild	20%	19%	21%
	Moderate	31%	24%	38%
	Severe	18%	14%	21%
HADS categories in depression subscale	Normal	53%	52%	54%
	Mild	17%	21%	13%
	Moderate	26%	21%	29%
	Severe	4%	5%	4%

Out of respondents who are or were treated by a psychiatrist, the most commonly drugs prescribed were selective serotonin reuptake inhibitors (16%). Table 4 shows which prescription medications were or are being taken by respondents. Greater improvement in wellbeing was reported by younger respondents (*r* = −0.22; *p* < 0.02; Spearman's rank-order correlation). There was no correlation between reported improvement in wellbeing after CBD use and: (1) frequency of CBD use, (2) amount of CBD dose taken, or (3) form of CBD use.

**Table 4:** Psychiatric medications taken by respondents.

	**Substance**	***n***
Medications taken by respondents in the past	Selective serotonin reuptake inhibitors	16%
	Serotonin and norepinephrine reuptake inhibitors	12%
	Trazodone	6%
	Opipramol	4%
	Alprazolam	3%
	Hydroxyzine	3%
	Pregabalin	3%
	Monoamine oxidase inhibitors	2%
	Quetiapine	2%
	Aripiprazole	1%
	Lamotrigine	1%
	Mianserin	1%
	Mirtazapine	1%
	Olanzapine	1%
	Risperidone	1%
	I do not remember the names	16%
	I have never received any prescription drugs from my psychiatrist	7%
Medications currently taken by respondents	Selective serotonin reuptake inhibitors	8%
	Serotonin and norepinephrine reuptake inhibitors	8%
	Pregabalin	6%
	Trazodone	3%
	Alprazolam	1%
	Aripiprazole	1%
	Quetiapine	1%
	Lamotrigine	1%
	Mirtazapine	1%
	Olanzapine	1%
	Opipramole	1%
	I am currently not using any prescription drugs	38%

The greater the reported improvement in wellbeing after CBD use, the lower the HADS score in both subscales (vs. HADS-A: *r* = −0.27; *p* < 0.01; vs. HADS-D: *r* = -. 26; *p* < 0, 01; Spearman's Rank-Order Correlation). A very strong correlation was found be-tween scores on the HADS Anxiety subscale and the HADS Depression subscale (*r* = 0.76; *p* < 0.0001; Spearman's Rank-Order Correlation).

Under the HADS Anxiety subscale, 69% of respondents qualified for the group that exceeded the norm criteria (score > 7 points), while in the case of the HADS sub-scale, 47% exceeded this cut-off point.

93% of the respondents did not observe any negative effects of CBD consumption. Two respondents reported the occurrence of anxiety disorders during therapy, while 1 respondent reported the following symptoms: depressed mood, addiction, diarrhea, xerostomia.

Only 17% of respondents reported that they were currently taking psychotropic drugs. This group is too small to perform a statistical analysis using the above statistical tests. 49% of respondents admit to having taken the above drugs in their lifetime. Individuals who admit to taking psychotropic drugs in the past are significantly more likely to trust psychiatrists (*p* < 0.0001; Mann Whitney U test).

A comparison was made between the groups of respondents divided into diagnosed/treated psychiatry and under-diagnosed/untreated psychiatry. Non-treated respondents were found to have significantly lower trust toward psychiatrists (*p* < 0.001; U Mann-Whitney test). Scores on the HADS anxiety and HADS depression subscales were significantly lower among psychiatrist-untreated respondents (p <0.001; U Mann-Whitney test). Nicotine use was significantly more common among psychiatric patients (42 vs. 22%; χ^2^ = 4.1; *p* < 0.05; chi-square test), while THC use was more popular among the untreated (49 vs. 30%; χ^2^ = 3.3; *p* < 0.05; chi-square test). Patients who did not receive psychiatric treatment were significantly less likely to consult a physician or pharmacist about CBD (32 vs. 2%; χ^2^ = 12.9; *p* < 0.001; chi-square test). There was no significant difference between groups in terms of: Frequency, dose and form of CBD use, age of respondents, reported improvement in wellbeing after taking CBD, use of alcohol, caffeine, psychostimulants and hallucinogens.

## Discussion

The path to the use of CBD in psychiatry is partially clear, as CBD has been approved by the U.S. Food and Drug Administration as a drug for the treatment of drug-resistant epilepsy, suggesting that the compound has a satisfactory long-term safety profile for this neurological condition (16). Data on the benefits of CBD in reducing the severity of depressive symptoms and anxiety are limited but promising. Some studies show that CBD is useful in treating depression, anxiety, sleep disorders and even problematic cannabis use, as well as in reducing the positive symptoms of schizophrenia, with little to no side effects such as diarrhea, which decreased over time (17, 18). Clinical studies are also encouraged by the authors of publications summarizing the achievements of science in the field of CBD use in psychiatry. They point out that studies in larger groups of people are necessary not only to determine the safety and usefulness of the substances in psychiatric treatment, but also to determine the efficacy of the treatment in the context of differences in symptoms of gender disorders, since most clinical trials have been conducted mainly in men (19, 20). There is still too many question marks to not monitor people who use CBD on their own. The situation in which patients decide to self-medicate their symptoms with a drug for which there is, for the time being, limited evidence of efficacy and safety is potentially dangerous because, apart from the side effects, such actions may worsen their mental state through the natural progression of depressive disorders, especially since some respondents choose to take more than CBD, including THC or hallucinogens, which may not be neutral among respondents.

The survey involved people of different ages (both minors and retirees), with different levels of education, and living in both rural and urban communities, which means that the use of CBD for self-treatment of depression is not limited to certain social groups. This information may be useful in further planning of scientific and educational activities in this area.

When analyzing the above responses, it should first be noted that only 19% of respondents consulted their doctor or pharmacist about taking CBD. At the same time, in the group of psychiatric patients, only 49% of respondents informed their psychiatrist about the use of CBD during psychiatric treatment. This situation is potentially dangerous because when patients buy CBD outside the pharmacy, this sale escapes the control of the pharmaceutical regulatory authority, which may encourage the accidental ingestion of other substances than intended, because when sales are outside the control of pharmaceutical regulators, consumers need to trust the honesty of the sellers. The situation of physicians and pharmacists being informed by the patient of the use of a psychoactive substance that is not an approved drug for the condition being treated is also extremely difficult. Categorical prohibition is unlikely to be effective, but it will limit the patient's honesty at subsequent visits, and acceptance of this state of affairs means that the patient accepts responsibility-at least in part - for the possible adverse effects of taking a psychoactive substance. The situation of physicians and pharmacists will not improve until they have accurate knowledge of the effects of CBD in various clinical situations and of interactions with the most common psychotropic drugs. There is an urgent need to complete this knowledge.

An important element in the mystery of the CBD phenomenon is the chemical composition of the oil itself or the dried fruit you buy. You should keep in mind that in addition to CBD and other cannabinoids, there are substances from other chemical groups, such as terpenoids, flavonoids, and alkaloids. It is possible that these substances may have an impact on the patient's wellbeing (21). It is important to know this because a possible complex antidepressant effect of Cannabis sativa- derived substances cannot be excluded. Research suggests that CB1 and CB2 receptors are associated with depression and bipolar disorder, and a single nucleotide polymorphism in the CB1 receptor has been observed in patients with treatment-resistant depression (22). CBD is an agonist of the 5HT-1A receptor, which in combination with its action on cannabinoid receptors may lead to a new unique effect (5).

As mentioned in the introduction, the media is eagerly interested in the topic of using CBD to treat depression, and society is picking up on the topic in social media. In public discourse, healthcare professionals should stick to facts. There is not enough data to conclusively confirm or rule out the claim that CBD is useful in treating mental illness. Given the social aspect of CBD use, further research by interdisciplinary teams made up of psychiatrists and pharmacists seems well warranted.

The responses collected shed light on another aspect. When planning further research on the use of CBD to improve symptoms of depressive disorders, it is important to pay attention to validated instruments that help in the diagnosis of depression. The responses to the question about reasons for starting CBD use may suggest that although we asked about self-treatment of depression, and this was clearly explained in the survey instructions and in the first question, some of the public may not fully understand the nature of this disorder. Patients could be suffering from major depressive disorder or mixed depression-anxiety disorder, and since it makes a difference in terms of the proper medical solutions offered byphysicians, it may not make a difference to patients. They might just call both disorders “depression,” whereas according to our HADS-A and HADS-D results, anxiety is actually more prevalent in our study group. Differentiating the causes of depressive disorders on the basis of the currently used International Statistical Classification of Diseases and Related Health Problems will make it possible to reduce methodological errors, contribute to a more rapid resolution of scientific problems and avoid inaccuracy in providing data to other scientists.

### Study Limitations

This study is probably the only study to examine the extent of self-treatment of depression with CBD, but it is not free of limitations. The data was collected during COVID-19 pandemic, which could have an impact on the respondents wellbeing in terms of depressive and anxiety symptoms. Due to anonymity of the study, the study was anonymous and was not prospective, therefore, we could not explain if CBD actually helps people who use the substance. The survey was conducted over the Internet, which limits the ability to rule out respondent error in completing the survey and prevents intentional bias from being ruled out. A small group of respondents does not allow for indepth statistical analysis and it is not necessarily representative for the population; furthermore, the selection of the group depends on activity on the Internet. However, the exploratory nature of this study provides solid justification for further research and analysis in this area.

## Conclusions

Psychiatrists should be aware of CBD use in their patients during their daily practice, as CBD use can be found within people from all walks of life for self-treatment of depression due to depressed mood. Due to public interest, there is a need for education and research on the efficacy and safety of CBD use for mental disorders.

## Data Availability Statement

The raw data supporting the conclusions of this article will be made available by the authors, without undue reservation.

## Ethics Statement

Ethical review and approval was not required for the study on human participants in accordance with the local legislation and institutional requirements. Written informed consent from the participants' legal guardian/next of kin was not required to participate in this study in accordance with the national legislation and the institutional requirements.

## Author Contributions

GW: conceptualization, project administration, and visualization. GW and MS: methodology and software. GW and RP: validation and writing—review and editing. GW, IS, and MS: formal analysis, investigation, and writing—original draft preparation. GW and IS: resources. MS: data curation. PG and RP: supervision. All authors contributed to the article and approved the submitted version.

## Conflict of Interest

The authors declare that the research was conducted in the absence of any commercial or financial relationships that could be construed as a potential conflict of interest.

## Publisher's Note

All claims expressed in this article are solely those of the authors and do not necessarily represent those of their affiliated organizations, or those of the publisher, the editors and the reviewers. Any product that may be evaluated in this article, or claim that may be made by its manufacturer, is not guaranteed or endorsed by the publisher.
